# Chronic viral hepatitis: policy, regulation, and strategies for its control and elimination in Ethiopia

**DOI:** 10.1186/s12889-016-3459-1

**Published:** 2016-08-11

**Authors:** Fassil Shiferaw, Meketew Letebo, Abate Bane

**Affiliations:** 1 World Health Organization-Ethiopia, Non_communicable Diseases, Addis Ababa, Ethiopia; 2 Freelance Public Health Researcher, Addis Ababa, Ethiopia; 3 Gastroenterology and Hepatology, Addis Ababa University Medical School and Ethiopian Gastroenterological Association, Addis Ababa, Ethiopia

**Keywords:** Chronic, Hepatitis B, Hepatitis C, Screening, Treatment, Policy, Strategy, Rights, Integration, Ethiopia

## Abstract

**Background:**

Hepatitis B and C are silent killers not yet recognized as major public health challenges in many developing countries with huge disease burden. In Ethiopia, Hepatitis B is endemic with an average prevalence of 10.8 %, and the prevalence of Hepatitis C is 2 %. The prevalence of both infections, however, is likely to be underreported due to the lack of diagnostic facilities and appropriate surveillance systems. Ethiopia is also among the many Sub-Sahara African countries lacking a coordinated and systematic national response to chronic viral hepatitis.

The objective of this study is to examine the current level of response to viral Hepatitis B & C in Ethiopia with the aim to bring identified gaps to the attention of relevant stakeholders and policy makers.

**Methods:**

This cross-sectional qualitative study was based on semi-structured in-depth interviews with 21 key informants from health facilities, health offices, pharmaceutical companies, regulatory bodies, professional association and blood bank units. Participants were selected purposively based on their role in the national hepatitis response. The investigators also reviewed available policy and strategy documents, standards of practice and surveys, and paid visits to pharmaceutical premises to check the availability of antiviral drugs. Thematic analysis was employed to make sense of the data. During the data analysis process, all the authors critically read the materials, and data was triangulated by source, interpreter view and thematic perspective to ensure accurate representation and comprehensiveness, and validation of the interviewees’ responses. Once each investigator reviewed the data independently, the team reached a common understanding of the scope and contexts of the information attained. Data were subsequently reduced to key concepts, and case stories were taken with successive revisions. The key concepts were later coded into most basic meaningful categories. The World Health Organization (WHO) global hepatitis response framework was used to organize the analysis.

**Results:**

Ethiopia is in the process of preparing strategic plan and guidelines for viral hepatitis. However, the country still lacks the required partnerships, and resource mobilization as a national health response is limited. Community awareness on the disease transmission and its sequel is poor. Viral hepatitis screening services are not widely available except for the occasional mandatory medical checkups for work or travel purposes. Healthcare providers often take no further action after diagnosing patients with viral hepatitis due to lack of treatment guidelines and strategic frameworks for screening, diagnosis, and treatment. Besides, drugs that are effective in the treatment of viral hepatitis are not available, mainly due to regulatory challenges.

**Conclusions:**

Viral hepatitis and its disease burden are getting little attention in Ethiopia and many low-income countries. The levels of technical guidance and financial support from the international community are low. To date, the response to the infections in Ethiopia is patchy. Thus, the country needs to formulate policy and strategies in the areas of disease surveillance, risk group identification and screening, use of the birth dose of hepatitis B vaccine, and care and treatment. Improving availability of data on viral hepatitis, access to low-cost generic drugs and developing and dissemination of treatment guidelines are also critical. Leveraging the successful Health Extension Program for a hepatitis response, and exploring ways to learn from and integrate into the HIV/AIDS program should also be considered.

## Background

The hepatitis B and C viruses cause chronic viral hepatitis, a major global health problem responsible for 57 % of liver cirrhosis [1] and 75 % of primary liver cancer [2] cases, respectively. Around 2.2 billion people (over a third of the world population) worldwide have evidence of past or present infection with the viruses, and around 500 million of these are chronically infected, more than ten times those affected by HIV/AIDS [3]. Each year, chronic viral hepatitis results in around 1.3 million deaths from chronic liver disease and hepatocellular carcinoma (HCC) [4]. This death toll is comparable with the 1.5 million deaths caused by HIV and 1.2 million deaths resulting from either tuberculosis or malaria each year [5].

Hepatitis B is responsible for the chronic infection of 240–350 million people each year. Around 780,000 of these die of either cirrhosis or liver cancer [6, 7]. Similarly, Hepatitis C infects 170 million people each year and causes chronic infection and death in 130–150 million and 350–500,000, respectively [8].

Hepatitis B virus is endemic in Africa next to Asia, with a seroprevalence rate of between 8 and 20 % [6]. West Africa is particularly affected, with 95 % of the adult population showing indicators of previous hepatitis B virus infection while the regional hepatitis C prevalence is lower than hepatitis B at 5.3 % of the general population. The world’s highest prevalence is in Egypt (17.5 %), followed by Cameroon (13.8 %) and Burundi (11.3 %) [9]. Africa also has the highest hepatocellular carcinoma incidence rate in the world, partly due to a 5–25 % co-infection rate with HIV [10], which accelerates the progression to cirrhosis and hepatocellular carcinoma [11, 12].

In Ethiopia, there is a lack of nationwide representative data on hepatitis B and hepatitis C infections. It is, therefore, difficult to present the incidence, prevalence, and mortality rates accurately. A national survey conducted several years ago, and regional estimates have shown wide geographic and socioeconomic variation in hepatitis B prevalence, ranging between 5.7 and 10.8 % [13–15]. The incidence is higher in males with an evidence of past infection marker (HbcAb) seen in 50 % by 20 years of age, and 70 % at the age of 49 [16]. The seroprevalence of HbsAg is found to be as high as 14.4 % in blood donors [17]. Earlier hospital-based studies showed that hepatitis B accounts for 12 % of hospital admissions and 31 % of deaths in Ethiopian hospitals [18]. Studies also found that at least one of the hepatitis markers was found in 78 % of patients with hepatocellular carcinoma, 86 % of chronic hepatitis cases, and 88 % of cirrhosis patients in Ethiopia [19].

In Ethiopia, the overall seroprevalence of hepatitis C is estimated to be around 2 %, and infection is most commonly associated with contaminated medical instruments [20]. A recent study from northern Ethiopia showed the prevalence of hepatitis C to be three-fold among HIV-infected individuals [21]. There are no studies from the country on other high-risk groups such as drug injectors, prisoners, and homosexuals. In general, hepatitis B and C infections are responsible for the majority of cases of hepatocellular carcinoma and liver cirrhosis in Ethiopia [1]. However, despite the higher rate of progression to chronic complications with hepatitis C, hepatitis B is more significant due to the high prevalence [22].

In Africa and Asia, hepatitis B is often caused by prenatal transmission from mother to child or household transmission from a close contact during early childhood. In these settings, hepatitis C is commonly transmitted by sharing needles for drug use, needlestick injury, improper sterilization of medical equipment and transfusion of inadequately screened blood [23]. The clinical manifestations of both infections depend on the age of the patient, immune status, and disease stage. Similar to HIV, the long “symptom-free” period, which characterizes both infections, contributes to their spread. The risk of chronicity of hepatitis B is inversely proportional to the age at which the infection is acquired. Perinatal and early neonatal infections have a chronicity of 90 % while the rate for infections acquired during the first 5 years of life drops to 60 to 20 %. The rates drop to as low as 5 % for infections occurring during adulthood [24, 25].

Overall, hepatitis B infection accounts for 30 % of cirrhosis and 45 % of hepatocellular carcinoma [5, 26]. Hepatitis C has a higher chronic asymptomatic rate of 55–85 %, with 15–30 % developing liver cirrhosis within 20 years. The risk of hepatocellular carcinoma in persons with cirrhosis is approximately 2–4 % yearly [27]. Hepatocellular carcinoma accounts for around 25 % of liver cancer worldwide [28]. In Ethiopia, the mortality rate reported due to hepatocellular carcinoma is among the highest in the world at around 93.45 per 100,000 [27].

Given the magnitude and seriousness of the problem, many agree that prevention and control of viral hepatitis need a high degree of attention similar to HIV, TB, and Malaria by all stakeholders, including governments and funding agencies. And there have been calls by various agencies, associations and groups for an organized global response with well-articulated policies and strategies to address this largely forgotten problem. The World Health Organization has proposed a comprehensive approach by outlining four strategic axes to guide viral hepatitis response in member countries. In this study, we used this framework to examine the existing level of response in Ethiopia. The objective is to review the policy environment, strategy and implementation and regulation of hepatitis B & C prevention, care, and treatment activities and bring the identified gaps to the attention of relevant stakeholders and policymakers.

## Methods

### Study design

In this cross-sectional qualitative study, key-informant interviews were used to obtain data directly or indirectly on policy, strategy and service delivery for viral hepatitis in Ethiopia. The information is primarily collected from participants who are considered the main stakeholders of the multisectoral national viral hepatitis response. Separate semi-structured interview guides were used for each group of respondents. Interview data was supplemented with a review of available policy and strategy documents as well as surveys and standards of practice. In addition, the investigators paid visits to pharmaceutical premises to check the availability of antiviral drugs used for the treatment of viral hepatitis. Data collection was conducted between December 2014 and January 2015 by two of the authors using semi-structured interview guides. Participants were asked open-ended questions. The interviews lasted an average of twenty minutes and recorded as handwritten notes.

### Sampling & data collection strategy

The investigators interviewed diverse groups of respondents, including policymakers, health care providers, patients, regulatory body officials, national and regional blood bank unit staff, laboratory technologists, pharmacists and staff from public and private pharmaceutical suppliers. The informants are categorized into six groups, with each group containing one to three respondents. In total, 21 people were interviewed as shown in Table 1. The first group represented staff from the Federal Ministry of Health (FMOH) and Drug Administration and Control Authority, who are responsible for policy formulation and strategy development. They provided insights on current policies and strategies regarding viral hepatitis prevention, care, and treatment. The specific issues addressed included availability and registration of antivirals for hepatitis treatment on essential drug list and matters related to treatment protocols for viral hepatitis. The information obtained from these was further substantiated through an interview with a committee member of Ethiopian Gastroenterological Association.

**Table 1:** National policy, strategy, guidelines and survey documents

1.	Health Sector Development Program IV 2010/11 – 2014/15, Federal Ministry of Health Ethiopia(FMOH)
2.	Ethiopia National Expanded Program on Immunization, comprehensive multi-year plan 2011–15 (FMOH-Ethiopia).
3.	Ethiopian National health care quality 2016–20, FMOH-Ethiopia
4.	National Essential Medicine List Fifth Edition, Food, Medicine and Healthcare Administration and Control Authority of Ethiopia (FMHACA).
5.	National blood transfusion services strategy, Federal Ministry of Health Ethiopia(FMOH) February, 2005,
6.	Ethiopia Global Health Initiative Strategy, The U.S. Ethiopia GHI Team, v. 4, December 22, 2010.
7.	WHO-Ethiopia country cooperative strategy 2012–2015.
Guidelines	
1.	Standard treatment guidelines for primary hospitals, January 2010, Food, Medicine and Healthcare Administration and Control Authority of Ethiopia (FMHACA)
2.	Standard Treatment Guidelines for General Hospital, 3rd edition, 2014, Food, Medicine and Healthcare Administration and Control Authority of Ethiopia (FMHACA).
3.	Standard Treatment Guidelines For health centers, 2nd edition, 2014, Food, Medicine and Healthcare Administration and Control Authority of Ethiopia (FMHACA).
4.	Infection prevention guideline for health care facilities in Ethiopia, July, 2004 Federal Ministry of Health Ethiopia(FMOH)
Surveys	
1.	Ethiopian National Hepatitis B Study. Kefene H^1^, Rapicetta M, Rossi GB, Bisanti L, Bekura D, Morace G, Palladino P, Di Rienzo A, Conti S, Bassani F, et al. J Med Virol. 1988 Jan;24(1):75–84.
2.	Evaluation of injection safety & health care waste management in Ethiopia, 2009 survey report, USAID, Ethiopia

The second group represented clinical practitioners who are responsible for diagnosing, treating and care of patients with viral hepatitis. The clinicians were recruited from Gastroenterology specialty centers in Addis Ababa, from a tertiary referral hospital in the capital and private clinics. Ethiopia is a country with more than 90 million people. However, it has a limited number of gastroenterologists and specialty services. For instance, the number of gastroenterologists in 2008 was only ten. Although the number of Gastroenterologists rose to 20 by 2015, it remains low for such a huge population. Besides, the number of senior gastroenterology consultants is very few, and their services are limited to the capital. Furthermore, there are only two Food, Medicine, and Health Care Administration and Control Authority (FMHACA) registered gastroenterology specialty private clinics in the country. Two of the senior gastroenterology consultants, including one working in FMHACA registered clinic, were purposefully selected for the interview due to their long experience, the fact that they see most of the referred cases and their knowledge of the trends in viral hepatitis epidemiology and diagnosis and treatment services in the country. The other clinical practitioners, an internist and general medical practitioners selected from public and private hospitals were asked questions regarding the tools they have and the challenges they face during screening, diagnosis, treatment and care of patients with viral hepatitis. The specific issues discussed include the availability of diagnostic facilities, treatment protocols, and drugs, and patient satisfaction.

The third group of interviewees were pharmacists from private and government-owned institutions. The pharmacists were asked about their knowledge and experiences with drug treatment for viral hepatitis, the rules and regulations on the handling of antiviral drugs, and the availability of medicines in the pharmaceutical market. The fourth group represents patients with chronic hepatitis, selected randomly from government and private-owned institutions in Addis Ababa. Emphasis was given to their real experiences regarding their diagnosis and treatment. They were also asked about their awareness of viral hepatitis before and after diagnosis, and the availability and affordability of the services. The fifth group represented lab technologists from national and regional blood bank units and general laboratory services. These participants were interviewed about laboratory aspects, including the screening protocol for hepatitis B and C infections. The last group are informants from pharmaceutical importing companies who gave insights on challenges associated with the rules and regulations surrounding the importing and distribution of hepatitis B vaccine and antivirals for the treatment of viral hepatitis.

In addition to the interviews, the available policy, strategy, survey and standard of practice documents were collected by interviewers in hard and soft copy formats during each institutional visit for the interviews. Additional materials were gathered through cross-referencing from the Internet (Table 1). Furthermore, the investigators made on-site check-up visits at four governments and six private pharmacies to check the availability of antiviral drugs used in the treatment of viral hepatitis.

### Data analysis

Thematic analysis [29] was used to make sense of the data. Interview notes and the strategic documents collected were thoroughly reviewed for themes relevant to the viral hepatitis response in Ethiopia. During the data analysis process, all the authors critically read the materials, and data was triangulated by source, interpreter view and thematic perspective to ensure accurate representation and comprehensiveness and validation of the interviewees’ responses. Once each investigator reviewed the data independently, the team reached a common understanding of the scope and contexts of the information attained. Data were subsequently reduced to key concepts, and case stories were taken with successive revisions. The key concepts were later coded into most basic meaningful categories. Through this process, 32 key concepts were coded, and the information gathered from participants, document reviews, and observation was categorized under the WHO’s four-axes global hepatitis response framework shown in Table 2 to help highlight the gaps in the health system.

**Table 2:** Thematic analysis framework and Interview matrix

AXIS I	Raising awareness, promoting partnerships & mobilizing resources	AXIS II	Evidence-based policy & data for action
Interview with policymakers^a^ Interview with gastroenterological association^b^ Interview with clients^e^		Interview with policymakers^a^ Interview with gastroenterological association^b^	
AXIS III	Prevention of transmission	AXIS IV	Screening, care & treatment
Interview with policymakers^a^ Interview with laboratory technologists^f^		Interview with health care professionals^c^ Interview with pharmacists^d^ Interview with pharmaceutical suppliers^g^ Interview with clients^e^	
In all four axes available national policy, strategy and research documents reviewed			

Note

^a^One from Federal Ministry of Health Disease prevention and control & one from Ethiopian Food, Medicine and Health administration and control Authority

^b^Executive member of Gastroenterologists association

^c^Two gastroenterologists, one internist and one General Practitioner from government and private health institutions

^d^Two participants from government and two from private pharmacies

^e^Two patients from government hospitals and two from private hospitals

^f^Four informants: Two from national and regional blood bank units and two from general lab units representing public and private sectors

^g^Two individuals from pharmaceutical suppliers interviewed

The interview questionnaires were reviewed by “Adera Gastroenterology Clinic” ethics committee and a waiver was given. Verbal consent was obtained from each interviewee before interviews commenced. Participants were assured anonymity and confidentiality of their responses.

## Results and discussions

Table 3 provides a summary of coded responses from the interviews for each of the six key informant groups along the four axes. Details of the findings are organized in a similar manner and discussed in the subsequent sections.

**Table 3:** Summary of interview data per informant group

AXES	Informant group	Coded responses
AXIS I	Policy makers & Gastroenterology Association informant	• Other competing priorities to get policy attention & budget allocation
Raising awareness, promoting partnerships and mobilizing resources		• No implementing & donor agencies
		• No organizing body
		• Minimal media awareness initiatives
	Patients	• Traditional beliefs
		• No awareness about the disease & transmission
		• Awareness obtained via incidental diagnosis of oneself or close relative
AXIS II	Policy makers & Gastroenterology Association informant	• No policy & strategy for prevention, care & treatment
Evidence-based policy & data for action		• No current national survey on both hepatitis B & C
		• No hepatitis surveillance system
		• No antivirals for hepatitis treatment registered on essential drug list
AXIS III	Policymakers	• Hepatitis B vaccine as pentavalent vaccine in Expanded Program on Immunization(EPI)
Prevention of transmission		• 24-h-Birth-dose of hepatitis B vaccine not available
		• No Adult Hepatitis B catch-up vaccine administration policy
		• No Hepatitis specific health promotion or health education strategy
		• HIV prevention activity indirectly helps in control of viral hepatitis
	Laboratory technologists	• Standard of Practice to screen HIV, Hepatitis, & C, and Syphilis in blood banks
		• Hepatitis screening only in private institutions upon request
AXIS IV	Healthcare professionals	• Hepatitis screening only in private institutions upon request
Screening, care & treatment		• No organized treatment facility in the country
		• Smuggled hepatitis treatment drugs are not affordable
		• Lab investigation for treatment follow-up done abroad through agents, and is neither affordable nor accessible to the majority
		• Patients neglected & discriminated
		• Getting treatment is a human right issue
	Pharmacists	• Antivirals for hepatitis treatment not available on market
		• Antivirals for hepatitis treatment not registered on essential drug list
	Drug suppliers	• No legal support to import antivirals for hepatitis and HIV
		• Case-based drug import by prescription is very expensive
		• No awareness as a major health problem
	Clients	• No awareness about hepatitis screening
		• Prescribed drugs not available on market
		• Smuggled hepatitis treatment drugs are not affordable
		• Feeling of discrimination
	Client case stories	• four case stories narrated

### Raising awareness, promoting partnerships, and mobilizing resources

#### Community awareness, beliefs, and taboos

One of the interviewees was a 60-year-old man who reported being healthy until experiencing progressive weakness and fatigue over the previous 2 years. He said he had hepatitis B and never heard of the silent course and seriousness the infection until he received the diagnosis. The experience of the other patients we interviewed was also similar with incidental diagnosis and a lack of knowledge on how they acquired it and the consequences.

The trade-off in viral hepatitis is the long duration of infection and complications. It often has a symptom-free clinical course which misleads around two-thirds of carriers not to seek medical care [30]. In Ethiopia, viral hepatitis is commonly known as “Yewef Beshita” in the widely spoken Amharic language, which means a disease from a bird, with the bird frequently associated being a bat. There is a widespread belief that if a bat flies around someone and drops its urine, a person gets liver disease, which manifests as a yellowish discoloration of the eyes and yellow-colored urine. The community does not understand viral hepatitis as a disease with asymptomatic stage or a carrier state. Those who have “Yewef Beshita” are traditionally advised to take bed rest, eat sweets, and go to a traditional healer for herbal medicine. It is generally considered as an illness with no modern treatment. This lack of awareness clearly puts the communities at risk. Hence, new hepatitis infections must be reduced as a priority by raising community awareness and providing access to appropriate and culturally acceptable information about the mode of transmission, its consequences, and currently available care and treatment to protect themselves, their families and others.

According to a participant from Ethiopian gastroenterological association, efforts are being made by the organization to improve community awareness. The interventions reported were broadcasting key messages on viral hepatitis through local radios and organizing world hepatitis day celebrations every year. The informant believed these efforts had contributed to raising community awareness and sensitizing policy makers to harness progressive policies and strategies. However, improving community awareness on viral hepatitis requires a well-defined and sustainable strategy with education given in school curricula, health facilities, and through regular campaigns to address existing knowledge gap and misconceptions. Given the similarity of hepatitis B and C infections with HIV, the possibility of addressing the issues during the HIV-related awareness raising efforts should be considered. In rural Ethiopia, community conversations contributed to successes in the national HIV/AIDS response by improving HIV testing uptake, patient support and treatment adherence [31]. Therefore, the authors believe that this might be a good lesson to replicate for viral hepatitis on awareness creation, community mobilization for changing risk behaviors and identifying vulnerable at-risk groups.

This study did not address the level of awareness among health workers in Ethiopia. Our literature review did not yield any studies comprehensively addressing the issue either. However, there is evidence that at least some precautions related to the prevention and control of viral hepatitis are not well-practiced by health workers in the country [32]. Conducting in-depth studies, and addressing the gaps through appropriate pre-service and in-service training would, therefore, be crucial for health workers. This will help health workers equip themselves with the necessary knowledge and skills to provide integrated prevention, care, and treatment services for patients and protect themselves from the infections through precautions and vaccination.

#### Promoting partnerships & mobilizing resources

According to the informant from the disease control unit of the FMOH, the organization had been working with local partners like the Ethiopian Gastroenterological Association, with which it had collaborated since its establishment in 2008. The association took part in capacity building, training, and advising, and had been active in the provision of current viral hepatitis messages and interviews to the media. The association's informant also reiterated the fact that the organization was instrumental in establishing the Ethiopian Liver Health Association (ELHA) and setting a global hepatitis network via the World Hepatitis Alliance. Besides, the participant from the ministry stated that the WHO country office had been actively working alongside the ministry in various areas that have a direct impact on prevention of hepatitis B and C infections. Areas of collaboration include blood safety and supply of vaccines for the expanded program on immunization.

Hepatitis B virus is a hundred times more infectious than HIV [33], and ten times more people have HBV infections [3]. Despite this, however, the global community paid much less attention to viral hepatitis. For instance, the budget allocated to it by WHO is 70 and 30 times less than that of TB and HIV, respectively [26]. In the recent past, we have witnessed a global coalition able to gather vast funding and policy attention on HIV, TB and malaria. This had contributed to a marked decline in deaths from these diseases over the last 13 years and helped Ethiopia and other resource-limited countries achieve the sixth Millennium Development Goal (MDG 6). Viable global partnerships with an integrated health system strengthening approach are vital for low-income countries like Ethiopia in responding to the multiple public health challenges they face. According to the participant from FMOH, however, no developmental agency was working in Ethiopia in the area of viral hepatitis prevention, care or treatment. Even the President's Emergency Plan for AIDS Relief (PEPFAR) addressed only those co-infected with HIV.

One of the most important partnerships would be working with leading organizations like WHO as it provides technical guidance in conducting a situational analysis and adapting strategies and guidelines to the country context. Ethiopia missed two opportunities to collaborate with WHO on viral hepatitis: the 2012 global baseline survey [34], and the civil-society survey [35]. Participation in these surveys would have provided valuable information for relevant policy formulation and designing strategies and programs.

### Evidence-based policy and data for action

Interview with policy makers from FMOH revealed that they recognized viral hepatitis as a public health agenda for a long time. However, the response had been slow due to “*competing priorities, lack of budget and a lack of partners providing support and guidance for the response.*” Currently, the ministry organized a national task force and started working on the development of strategic documents and treatment guidelines following the global momentum and international resolutions.

Despite the burden of viral hepatitis and the challenge it poses, chronic hepatitis was only recently fully acknowledged globally as a health problem. In 2010, the World Health Assembly declared its first resolution (WHA63:18) recognizing the condition as a public health priority and called member states to respond with a comprehensive approach to prevention and control [36]. The 2012 World Global Alliance survey showed that, out of 46 African countries invited, only twelve responded, and just two of these had national strategic documents on the prevention and control of viral hepatitis. Ethiopia was one of the countries that did not respond to this survey request [34]. Similarly, only 18 African countries responded to the 2014 World Alliance Civic Society survey [37].

A lack of hepatitis data is a global challenge not just limited to Africa. Most estimates used in articulating policies and strategies come from systematic reviews and statistical modeling. In Ethiopia, viral hepatitis is not included in the Integrated Disease Surveillance Response (IDSR) system, and a lack of periodic surveys contributes to the paucity of data. For instance, a national hepatitis B survey was conducted in Ethiopia three decades ago [13], and there has not been any nationwide survey on hepatitis C. Thus a few small-scale regional studies have been used to estimate the magnitude of the problem. Such studies clearly require careful interpretation to be used as a representative data for the whole country. Globally, the silent chronicity of the infection and a lack of adequate and timely data for clinical decision-making has contributed to the infection being neglected in the global public health agenda. The lack of data has affected viral hepatitis to the extent that it was not included in the Millennium Development Goals despite the huge disease burden. It is unfortunate deaths from the condition have increased by 50 % over the period covered by the MDGs [38]. Stephen Locarnini, director of the WHO Regional Reference Laboratory for Hepatitis B, expressed this mishap as “*an epidemiological oversight based on flawed data."* Furthermore, Charles Gore, President of the World Hepatitis Alliance, emphasized the continued lack of attention to the infections by stating that “*the failure is compounded by not including viral hepatitis in the post 2015 sustainable development agenda*” [38]. This neglect of hepatitis prevention, care, and treatment by the global community has negatively influenced developing nations like Ethiopia, and it is not surprising if such countries lack a coherent policy and strategy for a national response.

Data collection, analysis, and dissemination framework are prerequisites for informing policy and strategy in prevention and control efforts. Strengthening the surveillance system through a national cancer registry is also another step that could help quantify the scale and distribution of the disease and to measure and gauge prevention and control efforts. The FMOH Health Sector Development Plan (HSDP IV) in which little emphasis was given to viral hepatitis as a public health agenda is due to end in 2015. The upcoming plan (2016–2020) must provide strategic guidance on hepatitis in line with current global thinking.

### Prevention of transmission

Prevention of transmission primarily starts with behavioral change on safer sex. Beyond that, it includes institutional interventions that focus on prevention of mother to child transmission, safe blood transfusion, vaccination, and safe and rational injection practices and appropriate medical waste management.

#### Prevention of mother to child transmission

In sub-Saharan Africa, over 90 % of children with hepatitis B are infected early in life through vertical and early neonatal transmission from their mothers. In Ethiopia, the prevalence of hepatitis B in pregnant women is estimated to be around 3.7 % [39], and there is no screening protocol for these mothers during their antenatal visits. Countries like Cameroon, Mauritania, and Rwanda succeeded in developing national guidelines to prevent mother to child transmission of chronic hepatitis [40]. As a measure to improve maternal and child health, the Chinese initiative of integrating HIV, syphilis, and hepatitis B screening into antenatal care has been shown to be a cost-effective strategy [41]. However, universal screening for hepatitis C is not recommended [42] because of the low prevalence of hepatitis C, minimal risk of Mother To Child Transmission (MTCT), and a lack of cost-effective interventions to minimize the risk of transmission [43].

#### Blood screening

Interviews with national and regional blood bank laboratory technologists showed that screening for HIV, hepatitis B and C, and syphilis is routine as outlined in the blood bank standard operating procedure. The informants confirmed that the tests are serological and not molecular; thus, there is a risk of recent infection transmission in the window period. However, routine blood collection practice is from voluntary, non-remunerated donors, and blood collection from paid or replacement donors is not recommended by the standard to decrease the window-period risk.

Universal screening of blood and blood products before transfusion has long been a challenge in many countries due to several issues, including test kit shortage. Since 2001, the World Health Organization has been working in collaboration with PEPFAR and Center for Disease Control (CDC) to improve the availability, adequacy and safety of blood in Ethiopia. The country formulated blood transfusion strategy in February 2005. The strategy included the screening of blood and blood products for transfusion-transmissible diseases with particular emphasis on HIV, hepatitis B and C, and syphilis.

A few prevalence studies from Ethiopia have addressed blood donors. Blood donor screening in two regions of Ethiopia showed a 6.2 % prevalence of HBsAg and a 1 % prevalence of anti-hepatitis C infection markers [44]. Other similar regional studies showed a prevalence of hepatitis B in blood donors to be as high as 14.1 %, highlighting the need to strengthen hepatitis B and C screening practices among blood donors through regular quality control procedures.

#### Vaccination

According to the key informant from FMOH, Ethiopian hepatitis B prevention strategy, provided as a component of a pentavalent vaccine, has been in place since 2007. The activity was said to be supported by the Global Alliance for Vaccines and Immunization (GAVI). The participant also highlighted that the ministry had actively tried to implement a program for screening health care staff and providing them the monovalent hepatitis B vaccine, although with limited success due to budget constraints.

One of the greatest achievements in the prevention of hepatitis was the development of the hepatitis B vaccine in 1982. The vaccine is safe and 95 % effective against all genotypes [45]. Since 1991, the WHO has recommended universal hepatitis vaccination of children and high-risk groups to reduce new infections and minimize the progression to cirrhosis and hepatocellular carcinoma. The primary objective of vaccination is to reduce the disease burden of chronic hepatitis B infection and reduce deaths from liver cirrhosis and liver cancer. Globally, three different strategies are used to implement hepatitis B vaccination:

i. Prevention of perinatal infections via a 24-h-birth-dose vaccination; Routine childhood vaccination integrated as the pentavalent vaccine in the Expanded Program on Immunization;
ii. Catch-up vaccination in adolescents and high-risk groups.

### 24-hr.-birth-dose vaccination

Universal administration of hepatitis B vaccine at birth is not yet implemented in Ethiopia. In fact, only 10 % of newborns in Africa get the vaccine [46]. The World Health Organization recommends the birth dose of vaccination to be given in the first 24 h to prevent vertical and early neonatal transmissions. Therefore, introducing and scale-up of the birth dose in Ethiopia is critical as it prevents prenatal and early-childhood infections, which are the most common modes of transmission and have a 90 % rate of progression to liver cirrhosis and hepatocellular carcinoma. However, implementation of the vaccine requires routine screening of pregnant women, timely administration by skilled personnel, and certain medical technologies. These are some of the potential challenges which require careful planning and resource mobilization [47]. The other challenge is the possibility that GAVI’s support may not be available to Ethiopia as GAVI stopped its support for the monovalent hepatitis vaccine since December 2005 due to issues of cost effectiveness [48].

### Pentavalent childhood immunization

In Ethiopia, the pentavalent vaccine has been introduced since 2007, and within 7 years, a coverage of 84 % is reported [49]. The vaccine contains hepatitis B vaccine along with Diphtheria, Pertussis, Tetanus (DPT) and Haemophilus influenza B and is given at 6, 10, and 14 weeks of age in the Expanded Program on Immunization. The GAVI-funded primary preventive intervention through immunization is reported to reduce vaccine-preventable disease burden by over 80 %. Globally, the coverage of three-dose hepatitis B infant vaccination has reached 82 % [50], and countries are expected to achieve a coverage of ≥90 % nationally and ≥80 % in every district by 2020 [51].

### Monovalent hepatitis B vaccine

The World Health Organization recommends monovalent hepatitis B vaccination as a catch-up strategy in low and intermediate endemic countries [52]. In high burden countries like Ethiopia, it is expected that large-scale routine vaccination of infants reduces transmission. Even so, high-risk groups like healthcare professionals and waste handlers can benefit from this vaccination. However, the monovalent vaccine is currently expensive and inaccessible for the majority of those who need it. Thus, the government must encourage generic low-cost hepatitis B vaccination in the community as a family-based self-care approach.

Regarding vaccination for hepatitis C, the current availability of new second-generation Directly Acting Antivirals (DAAs), sofosbuvir, simeprevir, and their over 90 % cure rate for hepatitis C [46] is expected to underestimate the need for vaccination. However, issues of drug resistance, suboptimal efficacy for different genotypes, the high cost, and side effects justify the need for developing hepatitis C vaccine. Currently, the vaccine trial has reached the testing stage on an animal model which shows promise, and it is a public health priority [50].

#### Safe and rational injection practices and medical waste management

The key informant from FMOH believed the low prevalence of hepatitis C, especially in the post-HIV era, might be due to improved infection-control procedures in the health institutions and blood banks. Besides, infection prevention is incorporated into the pre-service training curricula of medical studies. This too, is expected to impact the incidence of viral hepatitis. There is, however, no documented evidence of what the interventions achieved so far in the country.

One of the positive spinoffs of the HIV epidemic on the health systems of developing countries like Ethiopia is improvements in infection prevention activities. However, a lot of work remains to be done in the area. A survey conducted in four regions of Ethiopia showed that 68 % of health facilities used inappropriate sharp waste disposal methods, and just 2 % of healthcare workers practiced appropriate recapping of needles [32]. In a knowledge assessment survey of health professionals about infections transmitted by needle-stick injuries, 98 % mentioned HIV while 75 % identified hepatitis B, and only 28 % mentioned hepatitis C. Similar study on waste handlers showed 95 % of them knew that needle or sharp materials prick can transmit infections. Currently, hepatitis B vaccination rate among healthcare professionals is 7 % [32]. A study in Addis Ababa on medical waste handlers showed that hepatitis B and C infections are more common among the handlers than non-handlers and, despite this, none of them received the hepatitis B vaccination [53]. A study from the Northern part of Ethiopia assessed the general infection prevention practice in a health care setting revealed that knowledge on infection prevention is around 84.5 % while only 54.5 % of the respondents had safe infection prevention practices [54]. Findings from these studies showed that despite enhanced knowledge of healthcare workers and waste handlers on the risk of infection from unsafe injections and improper medical waste handling, their actual practice is suboptimal. Therefore, the need to design and implement adequate preventive measures, including hepatitis B vaccination, and creating safe working environment should be emphasized.

### Screening, care, and treatment

In viral hepatitis infection, there are three main categories of cases: acute infection, chronic carriers and those presenting with complications. Early case detection, treatment, and care help prevent progression to serious complications, prevent premature deaths, increase productivity and reduce medical costs.

#### Screening

All in all, screening practice seems suboptimal in Ethiopia. One of the interviewees was a 40-year-old educated man working as a section head in a government office. When asked about the circumstances of his diagnosis, he replied: “*I did not know that I had hepatitis B until I was screened to go abroad for a short training course.*” He had a history of gastrectomy with blood transfusion in the early 80’s. Sadly, the health facility never gave him any further information, and he only became aware of the consequences and safety precautions when searching the web. He expressed his concern about the lack of routine screening practices by stating, “*I feel sad when I think about so many people like me who did not get the chance to be screened. And also those with the virus who are unaware of their status, and thus cannot protect their sexual partners*.” He appeared to have benefited from the screening as, after learning of his infection, his wife and two daughters were screened, found to be negative and obtain the catch-up hepatitis B immunization.

This case scenario and other interviews with clients showed that community awareness about hepatitis B and C infections in Ethiopia is very low, and that infections are commonly transmitted unknowingly. In Ethiopia, very few people have the opportunity to know their hepatitis infection status. Typically, people get tested either during pre-employment medical checkups, as a requirement for immigration, or during blood donation. The testing rate in Ethiopia is estimated to be about 10–15 % for HBV and 5–10 % for HCV [55]. According to WHO and World Hepatitis Alliance data, two-thirds of the world populations live in countries where there is no access to hepatitis testing [34]. Conducting mass screening for hepatitis B and hepatitis C might not be needed in Ethiopia given the high number of competing priorities and the large population size. However, targeted screening of high-risk groups such as health-care workers, medical waste handlers, commercial sex workers, people living with HIV, close contacts of hepatitis B and hepatitis C infected individuals, and hemodialysis patients can help prevent viral hepatitis in the most exposed.

#### Care and treatment

Physicians in private and government hospitals are frequently confronted with chronic viral hepatitis cases, including asymptomatic carriers and those with complications. Such individuals are usually identified during screening of clients for different purposes, after patients presented with complaints pointing towards liver problems, or as part of routine laboratory workups. All the interviewed health workers highlighted that they had a professional duty to treat hepatitis B and C patients based on current guidelines and recommendations by recent scientific evidence. However, they cited the lack of treatment guidelines and screening strategy and the unavailability and high cost of therapy as the major challenges to providing the necessary care to their patients. The frustration of health workers is evident in what one of the internists mentioned: “*I remember my clinical practice in the 90s when there were no antiretroviral drugs to treat HIV cases, and a similar scenario has happened to chronic viral hepatitis patients. I feel desperate because I am unable to help my patients with scientifically recommended modalities.*”

In recent years, advances in medicine have placed us in an era in which we can control hepatitis B and cure hepatitis C. A variety of drugs are currently available for the treatment of hepatitis B. These include Interferon alfa, PEGylated Interferon alfa, Entecavir, Tenofovir, and Adefovir [56]. Lamivudine is also used in many resource-poor settings because of its low cost, tolerance, and safety. Although its use as a single agent is diminishing due to the high rate of drug resistance, lamivudine still has a role in combination with Tenofovir in those who are co-infected with HIV [57].

For years, the standard treatment of hepatitis C infections has been a combination of Interferon and Ribavirin. This treatment protocol, given weekly, has a response rate of 40–50 %. A better response rate of 65–75 % was achieved with the arrival of DAAs Telaprevir and Boceprevir in 2011. The rates are even higher (>90 %) when these drugs are used in combination with the new second generation DAAs Sofosbuvir and Simeprevir. The second generation antivirals act against all genotypes of HCV, have fewer adverse effects and are used as a single pill dose treatment over a short course of 2–3 months [45].

Despite these promising advances in the treatment of viral hepatitis, several challenges remain. Access to these treatments and availability of systems for screening and virological monitoring remains low in many resource-poor countries like Ethiopia. Besides, treatment and accompanying laboratory tests are very expensive. In Ethiopia, for instance, the diagnostic package, including routine liver function tests costs between 220 and 425 USD, and drugs like Interferon cost as much as 7200 USD for a 6-month treatment. Furthermore, the services are limited to specialized clinics, which are very few and are found only in the capital.

Interviews with pharmacists working in private and government institutions confirmed that physicians commonly prescribe Interferon, Entecavir, and Tenofovir for chronic hepatitis B. These drugs are not available in any drug stores and pharmacies across the country. However, a few stores took orders from clients and obtained the drugs from abroad. Pharmacists interviewed from public sector told us that they had Lamivudine and Tenofovir on their shelves, but these drugs are only used for HIV-infected individuals free of charge. In addition, the authors visited ten pharmacies at different corners of the capital Addis Ababa to check the availability of antivirals used for the treatment of hepatitis B and the DAAs. None of the visited pharmacies had these medicines and vendors in most of the pharmacies were not familiar with DAAs.

Inability to use antiretrovirals for the treatment of hepatitis B is another aspect of the drug availability challenge. The experience of one of the authors clearly shows this problem and the frustration of patients who are aware of possible benefits from such drugs. The author witnessed a 32-year-old man who had hepatitis B and tried to get specific antiretrovirals from a hospital dispensary being labeled “dishonest” by a physician working in an NGO working mainly on HIV/AIDS. The doctor boldly stated, “*this man is trying to deceive us and get treatment from the HIV/AIDS drugs funded by PEPFAR.”* In this case scenario, the patient didn’t know the funding arrangements or the politics of HIV funding. He knew he could benefit from the use of such drugs and was genuinely attempting to get. This patient being considered a deceiver is appalling. Besides, irrespective of the funding arrangements, this kind of marginalizing attitude by health care professionals, the system as well by donor programs is unethical and raises human right and equity issues in health. This is also what one of the interviewed health workers discussed “*I know that chronic viral hepatitis, especially hepatitis B does not have a cure just like HIV, but current treatment modality prolongs the development of complications and gives an improved quality of life. I am curious that why programs and donors are not interested in combating viral hepatitis.*”

The Ethiopian Food, Medicines, and Health Care Administration and Control Authority (FMHACA) is responsible for regulating health care practice, premises, and health care products. According to the key informant from the authority, antiviral drugs like Lamivudine and Tenofovir are found in the national essential drug list for use in HIV/AIDS treatment only. Moreover, drugs like Entecavir are yet to be included on the list. The reason he gave for prohibiting the utilization of these medicines for hepatitis B patients is fear of misuse and the possible emergence of drug resistance. Donors also fund the drugs with an explicit agreement that they are strictly used for HIV program. However, the interviewee recognized the need to register effective antiviral drugs used for the treatment of viral hepatitis. As he said, the main challenge is a lack of policy and guidance from the Ministry of Health to support the process of including those medications in the essential drug list to be available outside HIV program context. Hence, the authority is currently providing on-demand temporary permissions through the drug registration department for individuals who bring prescription and few drug importers upon request. We confirmed this fact through our interview with respondents from drug importing company. As one of the participants stated, importers often get temporary need-based permission to import antiviral medicines for chronic viral hepatitis, and that they are not allowed to import such drugs in bulk. The informant also admitted that, as a business person, he was not interested in doing so, as the drugs are extremely expensive, and the turnover is very low.

Local governments, international donors, civic societies, and the pharmaceutical industries must work in partnership with the government to mobilize resources to ensure accessibility of low-cost generic antiviral drugs in low-income countries like Ethiopia. Alongside developing policy and strategies, and updating essential drug lists, the Ethiopian government should also work on identifying platforms for providing comprehensive services for viral hepatitis in an integrated way. In a country like Ethiopia, it is not usually possible to provide high-quality holistic specialty care throughout the country. However, the success achieved through an innovative Health Extension Program since 2003 at the primary health care level, made health care to reach the majority of the rural population. Thus, incorporating hepatitis activities in this institutional framework could facilitate meaningful gains in the coverage of such services. The possibility of integration with HIV prevention, care and treatment services is also one area for consideration. Given the similarities in mode of transmission, disease progress, some shared antiviral drugs, and the 5–20 % co-infection rate of HIV with either hepatitis B or C, using the HIV program platforms for a viral hepatitis response might prove cost effective. This allows sharing resources and helps avoid duplication of efforts from a health system strengthening standpoint. Some lessons from HIV treatment, such as the noble task shifting approach, can also inform future viral hepatitis care in rural Ethiopia.

In this study, we tried to show the existing challenges to the national viral hepatitis response in Ethiopia and highlighted gaps for policy and strategy attention. The study might have benefited more if there was some quantitative validation of the qualitative evidence presented here.

## Conclusions

Viral hepatitis and its disease burden are getting little attention in Ethiopia and many low-income countries. The levels of technical guidance and financial support from the international community are low. There is, however, a global momentum which is driving improvement in viral hepatitis response worldwide. To date, the response to the infections in Ethiopia is patchy, and the country lacks tools and systems necessary for an effective response at all levels. Thus, the country needs to formulate policy and strategies in the areas of disease surveillance, risk group identification and screening, implementation of the birth dose of hepatitis B vaccine, and care and treatment. Improving availability of data on viral hepatitis, access to low-cost generic drugs and developing treatment guidelines are also critical. Leveraging the successful Health Extension Program for a hepatitis response, and exploring ways to learn from and integrate into the HIV/AIDS program could facilitate the sharing of resources and help improve cost-effectiveness. Above all, providing care and treatment for people infected with viral hepatitis is a moral, ethical, and human rights issue that needs to be addressed promptly.

## Abbreviations

CDC, center for disease control; DAA, direct acting antivirals; DPT, diphtheria, pertussis, tetanus; ELHA, Ethiopian Liver Health Association; FMHACA, Food, Medicine, and Health Care Administration and Control Authority; FMOH, Federal Ministry of Health; GAVI, Global Alliance for Vaccines and Immunization; HbcAb, hepatitis B core antibodies; HbsAg, hepatitis B surface antigen; HBV, hepatitis B virus; HCC, hepatocellular carcinoma; HCV, hepatitis C virus; HSDP, Health Sector Development Plan; IDSR, Integrated Disease Surveillance Response; MDG, Millennium Development Goals; MTCT, mother to child transmission; NGO, Non-governmental Organization; PEPFAR, President's Emergency Plan for AIDS Relief; TB, tuberculosis; WHO, World Health Organization
