# Erratum to: Chronic viral hepatitis: policy, regulation, and strategies for its control and elimination in Ethiopia

**DOI:** 10.1186/s12889-016-3745-y

**Published:** 2016-10-10

**Authors:** Fassil Shiferaw, Mekitew Letebo, Abate Bane

**Affiliations:** 1 World Health Organization-Ethiopia, Non_communicable Diseases, Addis Ababa, Ethiopia; 2 Freelance Public Health Researcher, Addis Ababa, Ethiopia; 3 Gastroenterology and Hepatology, Addis Ababa University Medical School and Ethiopian Gastroenterological Association, Addis Ababa, Ethiopia

## Erratum

After publication of the original article [1] it was brought to our attention that author Mekitew Letebo was incorrectly included as Meketew Letebo. The correct spelling of the name is included in the author list of this erratum and updated in the original article.
